# Tracking Hydroplasticization by DSC: Movement of Water Domains Bound to Poly(Meth)Acrylates during Latex Film Formation

**DOI:** 10.3390/polym12112500

**Published:** 2020-10-27

**Authors:** Sebastian M. Dron, Maria Paulis

**Affiliations:** POLYMAT, University of the Basque Country UPV/EHU, Joxe Marti Korta Center, Avda. Tolosa 72, 20018 Donostia-San Sebastián, Spain; dronsebastian@gmail.com

**Keywords:** latex, film formation monitoring by DSC, hydroplasticization, bound freezing water, free water, minimum film-formation temperature, *T*_G_

## Abstract

The film formation step of latexes constitutes one of the challenges of these environmentally friendly waterborne polymers, as the high glass transition (*T*_G_) polymers needed to produce hard films to be used as coatings will not produce coherent films at low temperature. This issue has been dealt by the use of temporary plasticizers added with the objective to reduce the *T*_G_ of the polymers during film formation, while being released to the atmosphere afterwards. The main problem of these temporary plasticizers is their volatile organic nature, which is not recommended for the environment. Therefore, different strategies have been proposed to overcome their massive use. One of them is the use of hydroplasticization, as water, abundant in latexes, can effectively act as plasticizer for certain types of polymers. In this work, the effect of three different grafted hydroplasticizers has been checked in a (meth)acrylate copolymer, concluding that itaconic acid showed the best performance as seen by its low minimum film-formation temperature, just slightly modified water resistance and better mechanical properties of the films containing itaconic acid. Furthermore, film formation monitoring has been carried out by Differential Scanning Calorimety (DSC), showing that itaconic acid is able to retain more strongly the water molecules during the water losing process, improving its hydroplasticization capacity.

## 1. Introduction

The new developments and increased use of waterborne paints can be traced back to a growing environmental awareness and development of strict environmental regulations. However, the growth of the waterborne coating market is driven by architectural applications, an area which does not have as high requirements for durability and strength as industrial applications. For industrial surfaces, solventborne coatings are still used twice as often as waterborne coatings [1]. The main reason for this imbalance is that waterborne paints still do not reach the properties of hardness, block and print resistance, the ability to withstand freeze-thaw cycles and dirt pick up resistance combined with a low film-formation temperature, as solventborne paints do. The main reason for the lower properties of waterborne coatings comes from the process of film formation and thus from the connection between the minimum film-formation temperature (MFFT) and the glass transition temperature (*T*_G_) of the binder polymer. On the one hand, for coating applications, the formation of a hard and scratch-resistant coherent film is essential, which needs a polymer with a high *T*_G_. On the other hand, it must be possible to obtain a film at ambient temperature, which requires a low MFFT. However, the *T*_G_ and the MFFT are directly related to each other. This is known as the “film-formation dilemma” and constitutes one of the greatest challenges in the future development of waterborne latex particles for coating applications [2].

One approach to overcoming this obstacle is to make coatings with polymers having a low *T*_G_ that are crosslinked during the film formation to obtain films with good mechanical and chemical resistance properties [3,4,5]. However, if the crosslinking occurs at an early stage of film formation or during the polymerization reaction, the development of good properties is inhibited [6,7]. The second method is the use of supramolecular chemistry. The crosslinking is obtained through noncovalent bonds, e.g., electrostatic interactions [8] or hydrogen bonding [9,10]. The drawbacks can be weak bonds and negative influence on the electrostatic interactions by the other compounds of the latex formulation, e.g., the surfactant.

However, the current and most extensively used approach to overcome the film-formation paradox is the use of temporary plasticizers, i.e., low-molecular-weight organic molecules, also called cosolvents, which are volatile organic compounds (VOC) [11]. Plasticizers can be seen as coalescing aids. They change the *T*_G_ and MFFT (i.e., they decrease it) by influencing the diffusion of polymer chains during film formation. More precisely, plasticizers allow a better diffusion of polymer chains inside the polymer particle and among different polymer particles [2,12,13]. This effect is only temporary because the VOC evaporate during film formation, restoring the initial *T*_G_ and leaving a hard polymer film behind. However, this leads to environmental pollution by the release of VOC to the atmosphere.

As a result, a goal of modern coating investigations is to find alternative temporary plasticizers. A well-known compound which can have such a temporal plasticizing effect is water. This behavior is known as temporary hydroplasticization and has already been a subject of intense investigation [14,15,16,17]. Water promotes the polymer diffusion rates by two mechanisms. The first one is a contribution to the polymer’s free volume due to its low *T*_G_ (136 K). The second one occurs in the presence of hydrophilic groups in the polymer chain, especially organic acids. The combination of water and organic acids lubricates the diffusion process [17]. These hydrophilic compounds have the task to distribute and bind the water until the final stage of the film formation. The difficulty is to find a compound that effectively distributes and binds the right amount of water. It has to be ample water in order to obtain plasticization, but the temporary hydroplasticizer (THP) should not bind so much water that the mechanical properties or chemical resistance of the final polymer film are destroyed. Particularly suitable for this task are compounds with polar groups. These can bind a certain amount of water depending on their nature. A quick assessment of the hydroplasticization capacity of a compound can be found through Barrie’s linear group additive method [15]. This method quantifies the theoretical amount of water a functional group is able to bind and is used to calculate the overall water uptake of a compound. It is important to keep in mind that this method only gives an orientation and that the water uptake by the THP is not the only factor having influence on the plasticization effect. A good distribution of this water is also of great importance for the plasticization. To quantify the hydroplasticization effect on polymers, Tsavalas and Sundberg [15] implemented a simple a priori prediction for the hydroplazticized *T*_G_ based on the Flory–Fox equation [18]. For this calculation only the dry state *T*_G_ of the used polymer (*T*_Gdry_), the *T*_G_ of water and the saturated weight fraction of water for the polymeric structure (x_water_, obtained through Barrie’s linear group additive method) are needed. With these parameters, the wet *T*_G_ (polymer swollen with water) for a given polymer can be calculated as *T*_Gwet_ = 1/[(x_polymer_/*T*_Gdry_) + (x_water_/*T*_Gwater_)]. The difference between the dry and the wet *T*_G_ shows the extent of plasticization promoted by water. It is important to point out what is meant by the fraction of water in this equation. As presented by Hodge et al. [19], the *T*_G_ of the polymer does not decay constantly with the amount of water present in the polymer, but it stabilizes at a certain value of water content and the *T*_G_ does not decrease further for higher increments of water content. This water content is what has been called before “saturated weight fraction”, but it has also been referred to as “bound water”.

In order to understand the mechanism responsible for hydroplasticization, DSC may help to understand how water interacts with the polymer chains [20]. When a DSC is carried out with a polymer in presence of water, two categories of water can be distinguished: free water and bound water. Free water is unbound water and gives in the DSC the same signals as pure water. Bound water is water restricted through the polymer chain and shows in the DSC a lower transition temperature than pure water [21]. Bound water itself can be split into two categories: freezing and nonfreezing. Freezing bound water has a great influence on the reorganization of the polymer chains and the amount of nonfreezing water depends on the chemical structure of the polymer matrix [22]. Bound water is mainly responsible for the hydroplasticizing effect, even if nonfreezing water seems to have a higher influence in this process. By DSC, free or unbound freezing water and bound freezing water present in a wet polymer sample can be distinguished by their crystallization temperature, which will also provide information on their domain size, as they supercool at different temperatures depending on their size and the hydrophilicity of the polymer close to it.

Therefore, theoretically the water domain size can be calculated from the temperature shift of the freezing bound water peak obtained from the DSC measurements. No information has been found in previous literature about the domain size of water during the film-formation process of polymer latexes. However, theoretical approaches were made by the oil industry to analyze the size of water domains inside oil samples, which can be used also for latexes. The oil industry is dealing with complex opaque and concentrated water in oil (W/O) emulsions that appear over the complete range of oil processing [23]. Thus, precise characterization of this emulsion is important for security and manufacturing reasons. A key point of these analytics is the characterization of the water domains or droplets in terms of size and distribution. Normally, optical methods are used to obtain this information, but due to the opaque character, only samples of diluted emulsions can be used. DSC measurements provide an interesting alternative allowing the measurement of undiluted samples in order to obtain the needed information from the crystallization process of water. The crystallization process is influenced by the structure of the surrounding oil or organic phase, leading to characteristic peak forms and shifts in the freezing temperature. The majority of parameters needed for the evaluation of these thermograms are specific for water. Due to this, the concepts for water/oil emulsions can be transferred to any other water/organic emulsion when the crystallization process is not changed significantly.

To use the DSC for the determination of the water domains/droplets size Clausse et al. [24,25] defined the following relation between the radius R of the water domain (droplet) and the (shifted) freezing temperature *T* in a W/O emulsion (Equation (1)):

(1) $$R^{3}=0.69\;\frac{3\dot{T}}{4\pi \int_{T_{m}}^{T}A\exp\left[-\frac{16\pi \;\gamma^{3}V_{s}}{3L_{m}^{2}\;ln^{2}\left(\frac{T}{T_{m}}\right)}\;\frac{1}{kT}\right]dt\;}$$

where $\dot{T}$ [K·s^−1^] is the scanning rate, *A* [s^−1^·m^−3^] is the pre-exponential factor in the expression of the nucleation rate, *k* [Nm·K^−1^] is the Boltzman’s constant, γ [Nm^−1^] is the interfacial tension between the undercooled water and the ice germ, *V*s [m^3^·mol^−1^) is the molar volume of the ice germ, *L*_m_ [Nm·mol^−1^] is the molar melting heat and *T*_m_ [K] is the melting temperature. The parameters γ, *V*_s_, *L*_m_ and *T*_m_ are specific for water, allowing us to assume that this relation can also be used for any water/organic emulsion, as far as the crystallization mechanism is not changed significantly by the organic phase [24,25]. This equation is based on the relation between the radius of the ice germ R* and the freezing temperature *T*:

(2) $$R^{*}=-\;\frac{2\gamma \;V_{s}}{ln\left(\frac{T}{T_{m}}\right)\;L_{m}}$$

Equation (2) shows clearly that the lower the freezing temperature *T*, at constant *T*_m_, the smaller the radius of the germ, which has influence on the nucleation rate, resulting in a smaller water domain. For a DSC latex sample at the same drying stage and with the same amount of water, a lower domain size results in an increased number of droplets and thus in a better water distribution and plasticization effect. However, this equation cannot be used as such, because it is not possible to determine directly the interfacial tension γ between the undercooled water and the ice germ. For this, normally the relation between the water droplet size and the temperature is estimated by optical methods like cryogenic electron microscopy. In the case of the latexes investigated during this project, this is not possible because the freezing bound water is inside the latex particle. However, taking into account the latex particle size of ~120 nm for the emulsions used, the water droplet diameter D_Z_ of ~ 1 nm for the bound freezing water seems to be a reasonable assumption. Due to this, it was decided to use the relation between DSC temperature shift and droplet size obtained from a W/O emulsion analyzed elsewhere, with water droplet diameters in this range. Díaz Ponce et al. [26] analyzed W/O emulsions with calculated droplets sizes in the range of 0.5–1.5 nm. The values obtained with this approach cannot be considered as absolute because of the numerous assumptions made, but the trends obtained will be considered, allowing a comparison between the latexes and the hydroplasticization effect in each of them.

In this work, initially three functional monomers able to produce hydroplasticization were chosen: methacrylic acid, itaconic acid and polyethyleneglycol methacrylate. They were individually incorporated to a polymethyl methacrylate-*co*-butyl acrylate (60/40%) that was not able to form films at room temperature. Initially the macroscopic effect of the incorporation of three grafted THPs on the MFFT, *T*_G_, mechanical properties and water absorption of the final films was analyzed in order to find the one providing the best film-formation abilities, together with the less detrimental effect on the final films. Then, the hydroplasticization process was analyzed by DSC. The effect that the different grafted THPs had was analyzed by carrying out film-formation monitoring DSC at different drying stages. By the quantification of the water domain sizes for latexes with different THPs, a better understanding of the hydroplasticization effect was sought. To the best of our knowledge, this is the first time that such correlation between water domain size and hydroplasticization effect is quantified. Furthermore, the water absorption process of dried films containing THPs was also analyzed by DSC. The water distribution in both processes (film formation process) and water absorption of dry polymer films was found to be different by wet DSC, which stands as a powerful tool to monitor hydroplasticization.

## 2. Materials and Methods

### 2.1. Materials

Technical grade monomers methyl methacrylate (MMA, 98% purity, Quimidroga, Barcelona, Spain), butyl acrylate (BA, 98% purity, Quimidroga), methacrylic acid (MAA, 99% purity, Sigma-Aldrich, Taufkirchen, Germany) and itaconic acid (ITA, 99% purity, Sigma-Aldrich) were used to synthesize the latexes. Polyethyleneglycol methacrylate (PEGMA, 5000 g/mol, 99% purity, Evonik, Essen, Germany) was used as macromonomer. Sodium dodecyl sulphate (SDS, 99% purity, Sigma-Aldrich) was used as surfactant to stabilize the droplets and ammonium persulphate (APS, 99% purity, Sigma-Aldrich) to initiate the polymerization. All the chemicals were used as received. Deionized MilliQ water was used as polymerization media.

### 2.2. Methods

#### 2.2.1. Synthesis of the Latexes

**Figure 1:**
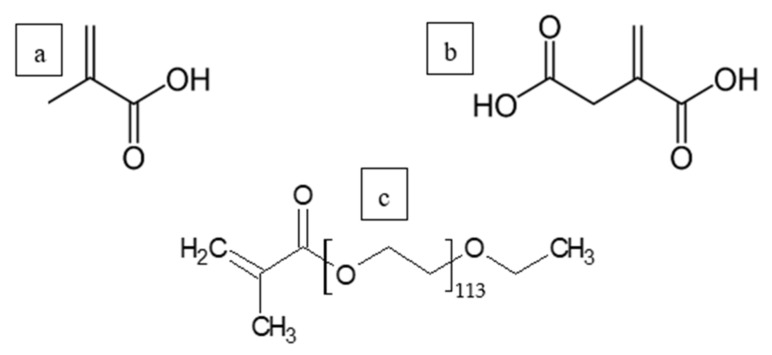
(**a**) methacrylic acid (MAA), (**b**) itaconic acid (ITA) and (**c**) polyethyleneglycol methacrylate (PEGMA) functional monomers.

Seeded semibatch emulsion polymerization was used for the synthesis of the latexes. The seed was prepared with BA/MMA (40/60 ratio) at 10% solids content with SDS as surfactant (0.5 wt % based on monomers) and APS as initiator (0.06 wt % based on monomers). Three different monomers were chosen to be used as grafted THPs: methacrylic acid (MAA), Polyethyleneglycol methacrylate (PEGMA) and itaconic acid (ITA) (Figure 1). MAA possesses a carboxylic acid group, the PEGMA a long ethylene glycol chain and ITA two carboxylic groups as hydrophilic moieties.

These functional monomers were fed together with BA/MMA at 40/60 weight ratio to the seed to produce the final latexes named with the name of the functional monomer (MAA latex, ITA latex and PEGMA latex) (Table 1). A reference latex was also synthesized, in which no functional monomer was included (REF latex). The reactions were carried out for 300 min at 80 °C.

#### 2.2.2. Characterization

MFFT was measured by a MFFT bar of our own construction. The steel bar was heated on the one side to 40 °C and cooled to 0 °C on the opposite side to produce a temperature gradient. The temperature along the bar was measured with PT-100 sensors. For the measurement, a polymer film with a thickness of 30 µm was applied on the bar. The temperature at which a coherent transparent film was obtained is the MFFT.

The mechanical properties of films dried at different temperatures were measured by tensile testing using a Stable Micro Systems Ltd. (Godalming, UK) with a constant velocity of 5 mm/s, corresponding to an initial strain rate of ca. 0.33 s^−1^.

The water absorption of the films was measured by immersing the films dried at 60 °C in water for 11 days. During this time, the samples were weighed every 24 h.

DSC measurements of the latex films dried at 60 °C for 24 h were measured on Q1000, TA Instruments (Hüllhorst, Germany). The scanning cycles consisted of first cooling to −20 °C at 10 °C min^−1^ (isothermal for 2 min), then heating to 100 °C (isothermal for 2 min), second cooling to −20 °C (isothermal for 2 min) at 10 °C·min^−1^ and then heating to 100 °C at 10 °C·min^−1^ and cooling again to 25 °C. The second heating cycle was used for the determination of the *T*_G_.

Apart from these DSC measurements of dried films, in this study DSC measurements of wet samples were also carried out. For the monitoring of the drying process, 5 mg of latex was weighed into the pan and we waited until a sufficient amount of water evaporated to obtain the desired water content. For these wet DSCs, the temperature range used was −60 to 60 °C and the heating rate 5 °C/min.

## 3. Results and Discussion

### 3.1. Initial Evaluation of the Hydroplasticization

Due to their flexibility, acrylic latexes are broadly used in the coating industry [27]. For all latexes used during this work butyl acrylate (BA) and methyl methacrylate (MMA) were used as basic co-monomers. By changing their relative monomer composition, the copolymer properties such as *T*_G_, chemical resistance and mechanical properties can be tuned [28,29]. The co-monomer ratio was set at 40/60 - BA/MMA in the REF latex, in order to obtain a *T*_G_ = 30 °C. Apart from the REF latex, three latexes in which MAA, ITA and PEGMA functional monomers had been added were also synthesized. The functional monomers were chosen based on the ability to be incorporated into the polymer chain and to bind water. The main goal was to identify a compound that is able to bind enough water to obtain a good plasticization effect without having a negative impact on the latex viscosity and on the film properties. Therefore, beside *T*_G_ and MFFT, the mechanical properties and the water absorption were investigated and compared.

All the reactions were carried out as seeded radical emulsion polymerization in a semi-continuous manner, in order to obtain 50 wt % solids content latexes. Final conversions higher than 99.5% were obtained. It has to be pointed out that the latex synthesized with ITA presented some challenges. ITA is a carboxylated water-soluble monomer used occasionally as co-monomer in the latex production by emulsion polymerization to improve colloidal and mechanical properties [30]. However, the emulsion polymerization of ITA with persulfate initiators is not straightforward. The reason is that ITA induces a decomposition of the persulfate radical, as described by Vanderhoff et al. [30]. The decomposition takes place through an electron abstraction from the ITA followed by rearrangement of ITA. Therefore, in order to obtain high monomer conversions as in the case of the rest of the latexes, a second shot of initiator was needed after the feeding of the first half of the monomers in this case.

Table 2 shows the MFFT and the *T*_G_ for the synthesized latexes. As reference, REF latex (BA:MMA—40:60, with 50% solids content) was used with a *T*_G_ of 30 °C and a MFFT of 25 ± 1 °C. The wt. % of the THPs used is based on the polymer content in each latex. Methacrylic acid (MAA) was the first grafted THP investigated because it has the potential to bind water and is known to readily incorporate into BA-MMA polymer chains [29]. However, the latexes containing MAA did not sufficiently show the desired effect as the MFFT was not significantly decreased. The used PEGMA grade (PEGMA 5005) showed a very good performance. The *T*_G_ was only slightly influenced and the MFFT range was decreased by 9 °C to 16 ± 1 °C.

The latex containing ITA showed a huge decrease of the MFFT down to 14 ± 2 °C, what is 16 °C lower than its *T*_G_. The wet *T*_G_ is calculated based on the water percentage being able to absorb each copolymer (based on Barrie’s method) and, with the aid of the modified Flory–Fox equation proposed by Tsavalas and Sundberg [15], the values present in Table 2 were obtained. It can be noticed that all wet *T*_G_s were lower when a THP was introduced in the copolymer. However, the decreases observed in the wet *T*_G_s were not completely correlated to the decreases in the MFFT values, as ITA latex showed a higher MFFT decrease compared to the slight wet *T*_G_ decrease. Therefore, it can be concluded that water absorption capacity obtained from Barrie’s method cannot be taken as the only indicator to evaluate the extent of the hydroplasticization effect.

**Figure 2:**
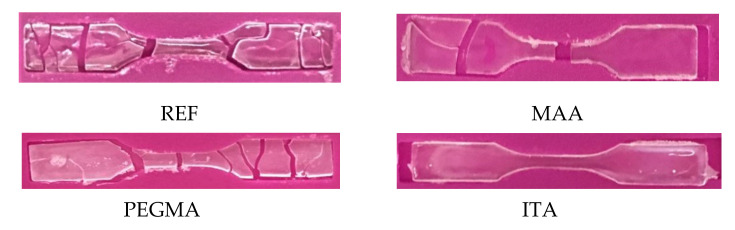
Specimens prepared for tensile tests dried at 20 °C, RH = 55% for 48 h.

The influence of the used THPs on the mechanical properties and the water uptake of the final dried films were also investigated. The films used for these studies were initially dried at 20 °C and relative humidity (RH) = 55% for 48 h. As can be seen in Figure 2, only ITA latex was able to form a continuous film for the tensile tests in these conditions. So, the hydroplasticization efficiency of ITA appeared not only as evidence of a decrease in the MFFT, but also as the capability of producing a continuous film at room temperature (of a polymer with a *T*_G_ of 30 °C).

**Figure 3:**
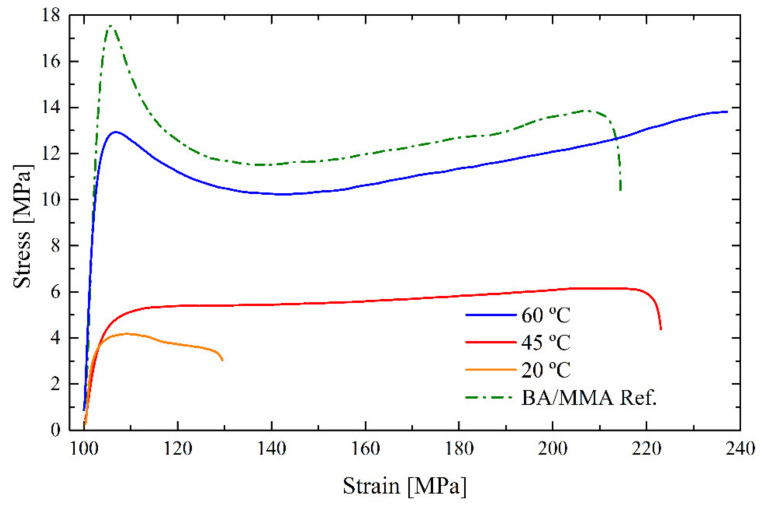
Stress–strain curves for films made with the ITA latex annealed 24 h at different temperature, and RH = 55%, compared to the reference (REF) latex dried at 60 °C.

The mechanical properties of the ITA film annealed at 20 °C could therefore be measured. As it can be seen in Figure 3, even if the film was continuous and transparent, its mechanical properties were not good enough. As a result, new ITA films were produced by annealing them at 45 and 60 °C for 24 h, after being dried at room temperature.

**Figure 4:**
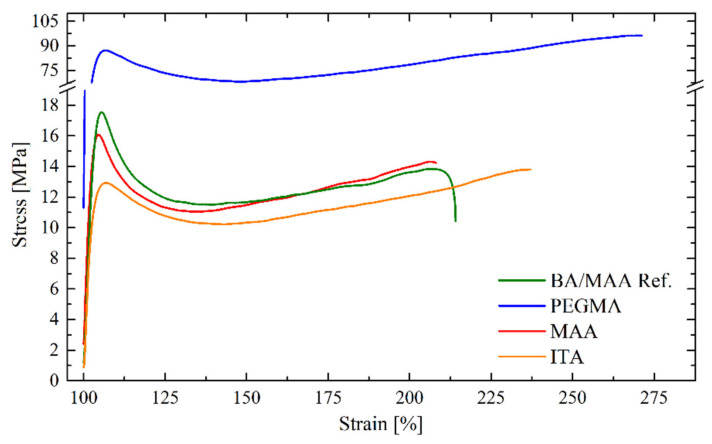
Stress–strain curves for the used films prepared with THPs annealed 24 h at 60 °C.

As expected, the mechanical performance increased with increasing the annealing temperature, at 60 °C reaching similar mechanic properties compared to REF latex annealed at 60 °C. If all the latexes with grafted hydroplasticizers were annealed at 60 °C, the mechanical properties shown in Figure 4 could be obtained. The films with MAA and ITA showed no notable difference with respect to the REF latex. However, PEGMA exhibited dramatically improved mechanical properties. In this case, even though the hydroplasticization effect, as seen in the decrease of the MFFT for PEGMA, did not allow production of continuous films at 20 °C, it can be seen that the final mechanical properties were improved when annealed at 60 °C.

**Figure 5:**
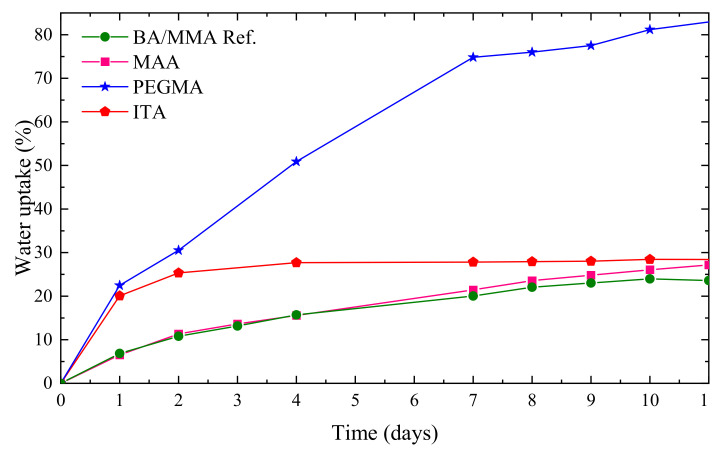
Water uptake curves for the films prepared with THP annealed 24 h at 60 °C.

Finally, the water absorption of the films with the grafted THPs was analyzed, for the films dried at 60 °C (Figure 5). PEGMA and ITA showed initially a strong increase in the water uptake compared with the reference latex, which means a strong decrease in the water resistance. However, after four days, the ITA film reached a plateau with the same water uptake of the REF latex (around 30%) while PEGMA latex continued absorbing water reaching more than 80% water uptake after 10 days. The latex containing MAA did not show any significant change in the water uptake compared with the REF latex. As a result, the incorporation of ITA as THP seems to be the most promising approach to obtain a temporary hydroplasticization, as it reduces the MFFT and produces cohesive films at 20 °C and the water resistance of the final dried latex is not seriously damaged.

### 3.2. DSC Measurements of Wet Samples

#### 3.2.1. Initial Wet DSC Measurements

**Figure 6:**
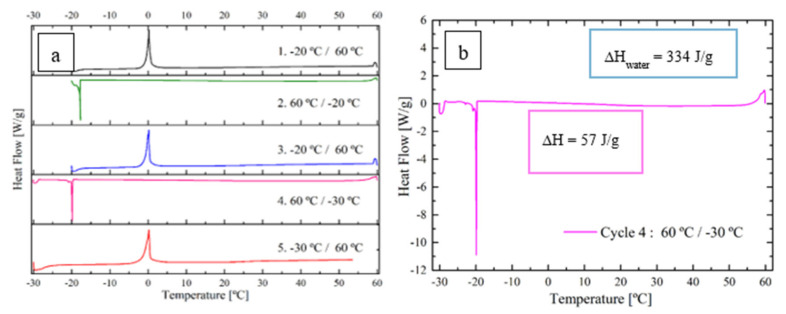
(**a**) Curves of the wet DSC experiment for PEGMA film (1.5 mg) with 1 mg of water. (**b**) Cycle 4 of the DSC experiment with the melt endotherm energy.

As explained in the introduction, in order to obtain a deeper knowledge of the hydroplasticization process, DSC measurements of THP latexes in the presence of water were carried out based on the work of Klots et al. [31]. There, the DSC measurements were carried out with dried resins to which a defined amount of water was added. The first series of experiments was conducted following the same procedure. The measurements were carried out with a dried latex film deposited with a weighed amount of water in an aluminum DSC pan. Figure 6a presents the thermograms obtained from this experiment carried out with the film obtained from PEGMA latex (1.5 mg) with 1 mg of water added to the aluminum pan. For every heating or cooling cycle the heat flow against the temperature is shown. The first three cycles are carried out to homogenize the sample with the water inside the aluminum pan. The cycle used for quantification and evaluation is the fourath one (Figure 6b), were the sample is cooled down from 60 to −30 °C.

Figure 6b presents the crystallization peak of nonbound freezing water at −19.4 °C, and another crystallization peak seems to start appearing at temperatures lower than −25 °C, which could be due to bound freezing water. However, due to the conditions in which this DSC was run, the crystallization of the bound freezing water could not be properly observed. However, from the melt endotherm energy ∆*H* obtained at −19.4 °C, the amount of nonbound freezing water can be calculated, and given the total amount of water added to the system, the amount of bound water can be inferred [23]. For the example shown in Figure 6b, where ∆*H*_DSC_ = 57 J/g _sample_ and knowing that the endotherm value for free water crystallization is ∆*H*_water_ = 334 J/g, taking into account that 1.5 mg of polymer and 1 mg of water were initially added to the sample, it can be concluded that 43% of the water initially present in the pan was not bound to the polymer (and it froze at −19.4 °C), and the rest (57%) was bound to the polymer. As a result, each polymer gram was able to bind 0.38 g of water, i.e., 27.5% of water in the water swollen polymer. Similar DSC experiments (dry polymer + liquid water added in the aluminum pan) were carried out for all the films produced with grafted THPs, in order to determine the amount of bound water (see Table 3).

It is, first of all, apparent that the calculated amounts are significantly higher than the amount of water predicted with Barrie´s linear addition method (see Table 2). This circumstance, however, was already observed by Klots et al. [31] and is caused by the fact that Barrie did not distinguish different categories of water. Nevertheless, the amounts of the bound water obtained by DSC correlate quite well with the macroscopic properties measured earlier. In fact, REF and MAA latexes showed similar MFFT and water uptake behavior (Figure 5). PEGMA film shows the highest water uptake in the water absorption test, and it is the one that shows the highest bound water content here as well. Finally, the ITA film initially also showed a higher water uptake than MAA and REF latexes, as shown by the DSC calculation of bound water. Furthermore, both PEGMA and ITA latexes showed a decreased MFFT compared to the REF latex. As a conclusion, a similar trend is observed between the hydroplasticization capability and the amount of bound water by DSC.

However, the appearance of the small signal around −25 °C in the thermogram in Figure 6b indicates that the temperature during the DSC was not low enough to observe a second water freezing peak, the one coming from bound freezing water. For this reason the experiments were repeated with a freezing cycle reaching −60 °C. The samples were prepared as before, with dry latex films immersed in water and measured directly after the preparation. The expectation was to obtain two peaks during the freezing cycle, one for the freezing free water and one for the freezing bound water in the area around −40 °C [15]. However, for all measurements the peak for the freezing bound water did not appear.

It is probable that the absence of this peak was caused by the sample preparation, more precisely by the lack of time allowed for the water to penetrate the sample deep enough. For this reason, the strategy for the sample preparation was changed and the new samples were prepared with a dry latex immersed in water for one week before the measurement.

#### 3.2.2. DSC Analysis after Immersion of Films in Water for One Week

**Figure 7:**
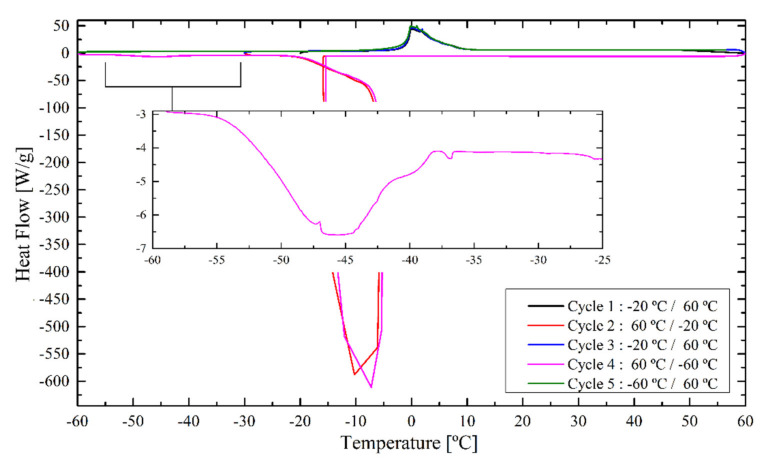
DSC measurement of the REF latex immersed for one week in water.

As an example of the results obtained with this new procedure, the thermogram for the REF latex immersed one week in water is shown in Figure 7.

**Figure 8:**
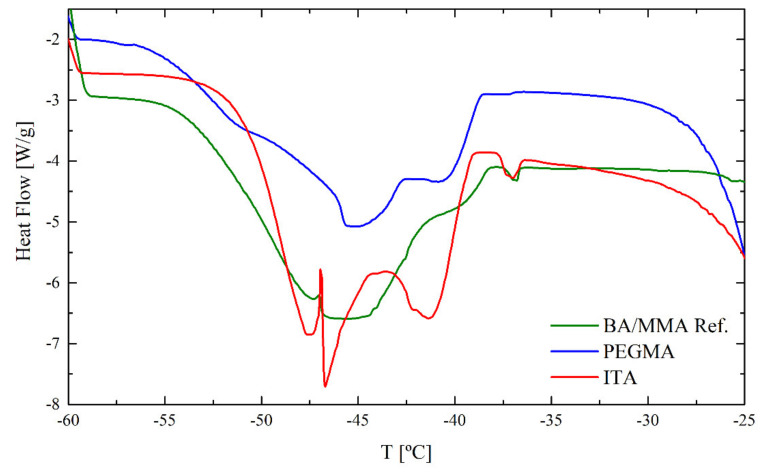
Freezing bound water peaks for the REF, PEGMA and ITA films immersed for one week in water.

In contrast to the results before, now a clear peak can be observed at −42.5 °C, corresponding to freezing bound water, apart from the one of the freezing unbound water. The peak becomes visible because of the increased time the water had to penetrate the polymer matrix and interact with it. The freezing bound water peak contains the most relevant information to study the process of hydroplasticization in conjunction with THPs. This is why the discussion will be focused now on these DSC peaks. Figure 8 shows the freezing bound water peaks for the different films immersed for one week in water.

For the REF latex, a broad curve can be observed centered at −45 °C. This means that the water is able to penetrate into the polymer film and distribute well along the polymer matrix. This film consists only of BA and MMA, without any functional grafted THP, which allows a statistically and regular water distribution throughout the polymer matrix. In the case of the latexes containing ITA and PEGMA, a different result can be observed. The functional monomers create areas of higher and lower hydrophilicity causing an irregular water distribution. In the case of the ITA film, two peaks can be clearly distinguished, one at −41 °C and the second one at −47 °C (split by an artifact). The bound water crystallizing at lower temperatures probably is linked to the more hydrophilic ITA moieties, while the bound water crystallizing at higher temperature is linked to the acrylic polymer without ITA. The same splitting, even if less expressed, can be observed for the PEGMA film, showing domains more and less hydrophilic.

These DSC measurements down to −60 °C after immersion of the films in water for one week allowed the calculation of the amount of freezing bound water, and by subtraction, the amount of nonfreezing water (knowing also the amount of bound water). The calculated amounts are shown in Table 4. The first aspect that can be mentioned is the increased amount of bound water after immersion of the films for one week in water, compared with the amounts of bound water present in Table 3. Furthermore, the amounts of water absorbed still match the water absorption gravimetric measurements present in Figure 5. Finally, it can be seen that the amount of nonfreezing bound water is significant for the three samples, even if it is slightly higher for the films containing the grafted THP moieties.

The quantification of the bound water for the samples immersed in water for one week provides insight to the water distributed in the final dried polymer film. This measurements could be of interest when studying the water uptake by latex containing different surface functionalities [32] or when analyzing the underwater adhesion properties [33]. However, this is a redistribution of water reentering into a formed polymer matrix of a dried latex film. For the actual hydroplasticization effect, the water distribution during the drying process is of greater importance. Therefore, DSC measurements were also carried out to monitor the film-formation process.

#### 3.2.3. DSC Analysis during Latex Drying

As mentioned in the previous section, DSC measurements were also carried out with latexes at different drying stages, taking the measurement down to −60 °C. Wet latexes were placed in the aluminum pans used for the DSC measurements, and their weight was monitored along time, in order to stop the drying step at different water contents, from the initial 50% water content (50% solids content) to the final 0% of water. The discussion of the obtained results will be focused on the freezing bound water peaks.

**Figure 9:**
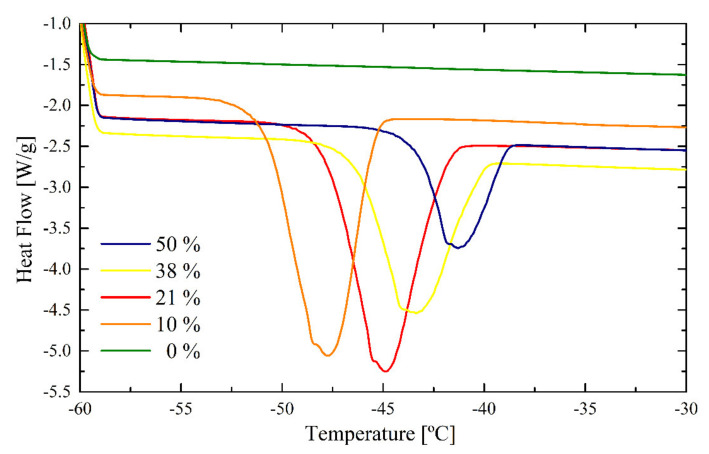
DSC peaks for bound freezing of the REF latex.

Figure 9 shows the DSC curves of the freezing bound water for the REF latex, where a shift of the crystallization peak to lower temperatures with decreasing water content can be observed. The absence of any THP functional monomer allows to observe a typical evolution of the freezing bound water peak during the drying process. The peaks have a Gaussian form with a single maximum, and its area increases from 50% water content to 21% water content, where it is maintained at 10% water, and disappears when the latex is completely dry (0% water content).

To understand the obtained thermogram, the crystallization process of water, especially of small water domains, has to be taken into account. To form a crystal structure, a certain amount of H_2_O molecules, which need to overcome a specific energy barrier is needed. In the case of water, this can happen through sufficient cooling until an ice nucleus is formed. Normally, water freezes around 0 °C. The nucleation is a stochastic process. The more water molecules are present, the higher the probability of nucleation and crystallization. In a small water domain, the probabilities are lower, and therefore a lower temperature is necessary. This phenomenon is known as supercooling. With progressive drying of a latex, the water domains become smaller, resulting in the observed crystallization temperature shift for different drying stages. It is also possible that the thermogram shows one curve with multiple crystallization peaks, in a process known as fractionated crystallization [34,35].

**Figure 10:**
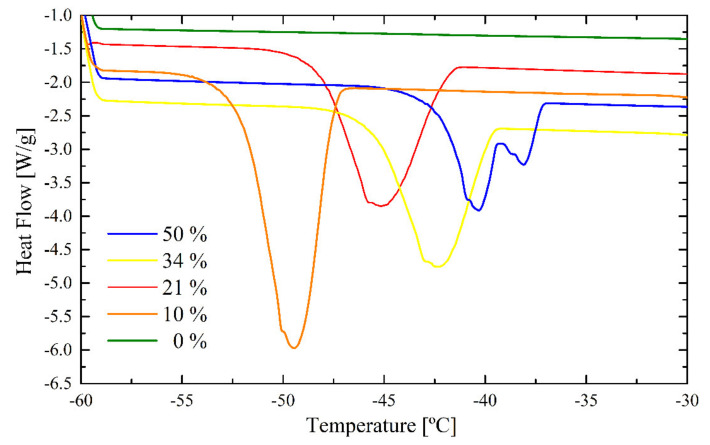
DSC peaks for bound freezing of the ITA latex.

Figure 10 shows the bound water DSC peaks for the ITA latex. For the samples with the highest water content, the not-dried latex, a splitting of the signal into two peaks can be observed, similar to the splitting observed for the films immersed in water for one week. This indicates the existence of two main polymer domains with higher and lower hydrophilicity and a good incorporation of ITA into the polymer matrix.

As for the REF latex, a peak shift down to lower transition temperatures and an increase of the peak area with decreasing water content can be observed. The biggest shift occurs for the sample with 10% water content. The water is distributed well throughout the polymer matrix in such small domains so that −50 °C is necessary to freeze it. This is the biggest shift of all analyzed samples.

**Figure 11:**
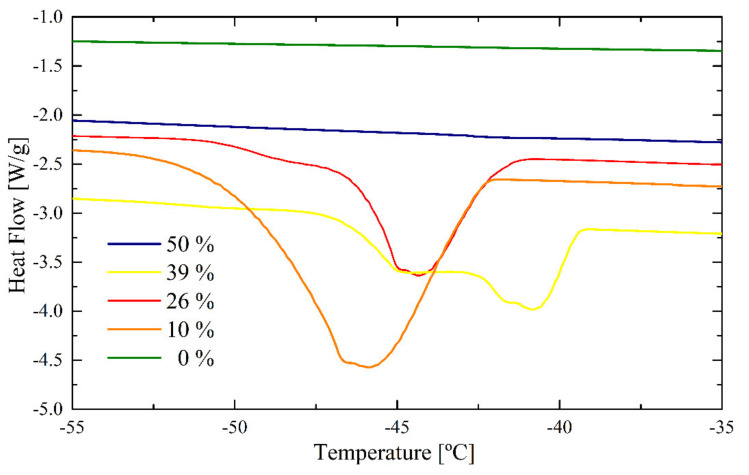
DSC peaks for bound freezing of the PEGMA latex.

The bound water peaks corresponding to the drying of latex containing PEGMA are shown in Figure 11. In the case of the wet latex before drying (50%, blue) no distinction between bound and unbound water can be made. The thermogram shows only one peak with a transition temperature around −15 °C (not shown in Figure 11). This means that all water present in the sample is in water domains of similar size, which freeze as unbound water. With decreasing water content crystallization peaks for “bound water” appear, which, in the case of 39% water content, are split into two bands, corresponding to water bound to moieties with different hydrophilicities. With advancing drying, a shift to lower transition temperatures and an increasing intensity can be also observed for the PEGMA latex.

The ITA latex and PEGMA latex both contain a THP, the hydrophilic functional monomer. This functional monomer disturbs the statistical distribution of freezing bound water observed for the REF latex by creating areas of higher and lower hydrophilicity. The results show that ITA allows strong binding of water into the polymer matrix until the last stage of the drying process, the stage where the chain interdiffusion occurs. This explains the strong decrease of the MFFT, down to 14 ± 2 °C.

To confirm the robustness of the new implemented procedure for monitoring the film drying by DSC, the measurements for the drying latexes were repeated for ITA, PEGMA and REF latexes, and it was found that the results are reproducible.

As it has been observed, the freezing bound water crystallization peaks found during the drying step of the latex are very different to the ones observed in the films immersed in water for one week. In fact, the process of water entering the dried film (important for water resistance) is different from the process of water leaving the latex during the film formation (important for the hydroplasticizing effect), what is essential for the plasticization effect. Therefore, the following of the drying process by DSC can be a useful tool to study the hydroplasticization effect. The determination of the water domain size has been proposed in this work as a mean to quantify the hydroplasticization process.

#### 3.2.4. Determination of the Water Domain Size by DSC

**Figure 12:**
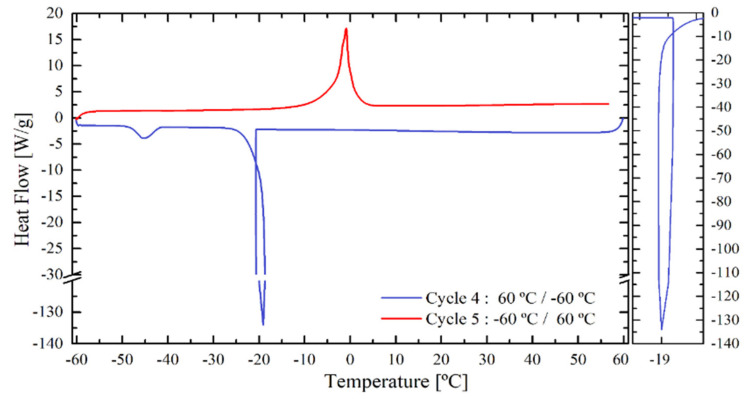
Freezing and heating DSC thermogram of the ITA latex with 10 wt % water.

Before calculating the water domain size, first the position of the water crystallization peaks will be discussed briefly to remind some conclusions discussed earlier. As an example, the thermogram of ITA latex with 10 wt % water content will be used. Figure 12 shows the DSC thermograms obtained in cycle 4 (cooling) and 5 (heating). Cycle 4 presents two crystallization peaks. The first peak occurring during the freezing is the one for pool/unbound water at −19 °C, a sharp peak characteristic of unbound water. As the amount of water is small and not restricted by the polymer, only one germ is sufficient for the nucleation, starting a fast crystallization leading to a big energy release in very short time. Furthermore, the free water shows a significant undercooling of −19 °C. Following the nucleation theory, the reasons for this undercooling are the size of the water domain and sample size, respectively. The smaller the water volume, the lower the freezing temperature *T*_Freeze_ (Table 5) [36].

The second peak that appears in the fourth DSC cycle around −45 °C is the one assigned to the bound freezing water. The higher shift compared to the prior peak, shows that this domain is significantly smaller. This is an important relation regarding the hydroplasticization effect, because the smaller the domains of bound water, the better the distribution in the polymer matrix leading to a better plasticization effect. Finally, the melting of ice is independent of the water size and location, so that the heating cycle 5 shows only one peak around −1 °C, as there is no delay in the melting process. The temperature slightly lower than the ice/water freezing point (0 °C) is caused by the presence of compounds dissolved in the water phase (surfactant, initiator etc.).

**Figure 13:**
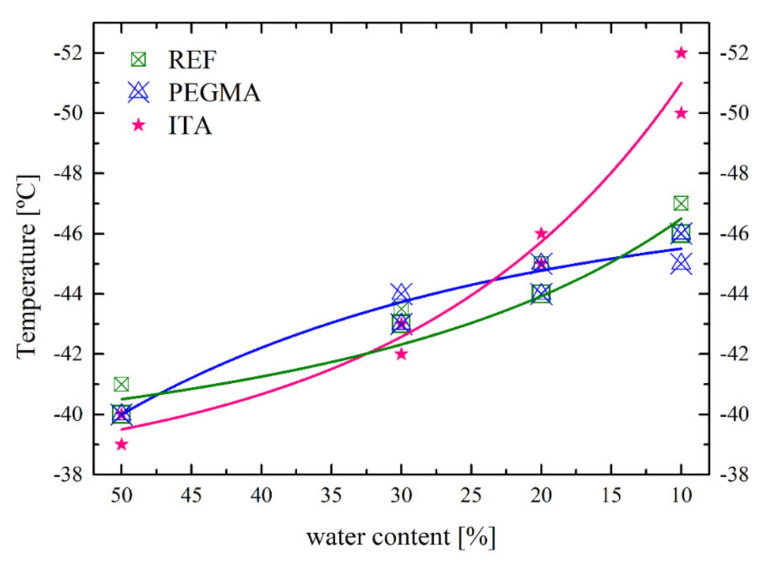
Freezing temperature of bound water for REF, ITA and PEGMA latexes during the drying.

Therefore, the distribution of water domains in a drying latex will determine the water domain sizes, which, as seen before, will determine the crystallization temperature. To draw conclusions, the freezing temperatures for the different drying stages were collected for repeated DSC measurements during the drying of the different latexes and were plotted in Figure 13. As freezing temperature, the apex of the curves was chosen. For double peaks the average of the apices was used. The fits to the data sets show, in all cases, an exponential slope corresponding to the trends observed by Clausse et al. [24,25]. The temperature shift also corresponds with the MFFT decrease and the plasticizer performances observed for the different latexes. Latexes containing ITA showed the lowest MFFT (14 ± 2 °C) and the biggest temperature shift in the thermogram.

**Figure 14:**
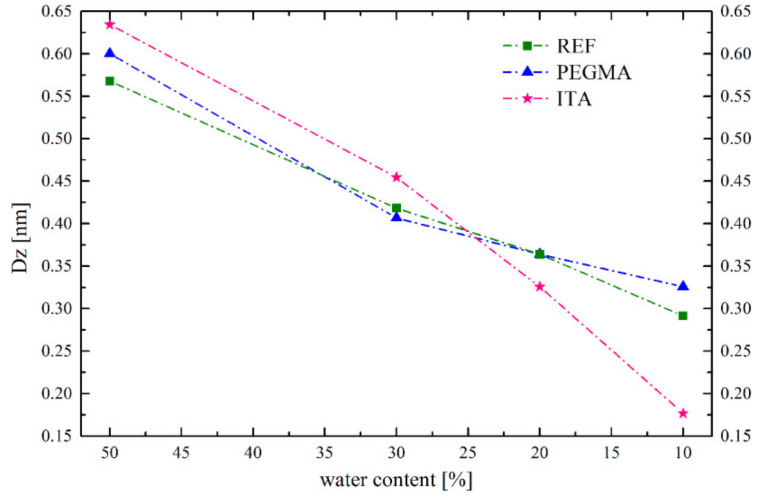
Water droplet size evolution, estimated form DSC measurements, over the latex drying process.

Using the freezing temperatures and the assumptions presented in the Introduction, the size of the bound water droplets was estimated and plotted versus the drying stage (Figure 14).

The calculated droplet size range from 0.17 to 0.64 nm seems small taking into account the size of water at around 0.27 nm [27]. However, as commented before, these values cannot be seen as absolute, but as relative. The trend observed in Figure 14 can be related to the MFFTs measured for the different latexes, as smaller droplet sizes (at 10% water content) coincide with lower MFFT (for ITA latex). Furthermore, if the initial droplet size is considered for each case, and a fivefold volume reduction is considered (going from a water content of 50% to 10%), the final droplet size could be calculated, considering that the number of droplets has not decreased, that they have just shrunk. If these calculations are done, final water droplet sizes of 0.33 nm for REF latex, 0.35 nm for PEGMA latex and 0.37 nm for ITA would could be envisaged. The values obtained for REF and PEGMA latexes correspond quite well with the final estimated water domain sizes, but the estimated water size value for ITA latex is much smaller than the one obtained from the shrinkage of initial droplets. This probably means that, in the ITA latex, bound water domains do not just shrink, but they are split apart forming more and smaller water-bound domains over the polymer matrix. The reason for this splitting is thought to be the high water-binding ability of ITA moieties, which is at the same time the ability that produces the hydroplasticization effect during film formation and a lower MFFT value.

In summary, it can be considered that monitoring the drying process of latexes can give an extensive set of information about the hydroplasticization process during the latex film formation process. It is not possible to obtain exact absolute numbers of water domain sizes, but accurate trends allow an accurate comparison between latexes and the influence of the functional groups introduced into the latex as THPs.

## 4. Conclusions

The effect of different compounds able to bind water to the polymer matrix and favor its hydroplasticization during the film formation process was analyzed in this work. Specifically, the goal of the temporary hydroplasticizers (THP) was to decrease the MFFT, without affecting the rest of the film properties. A BA/MMA (40/60) latex was chosen as reference latex, to which different grafted THPs were included. The results obtained from this investigation showed that the basic concept of temporary hydroplasticization works and can be tuned by incorporating the right functional compound. From the chosen grafted THPs (MAA, PEGMA and ITA), it was shown that with the addition of itaconic acid it was possible to decrease the MFFT down to 14 ± 2 °C without changing the *T*_G_, and with just slight impacts on the chemical resistance and improving the mechanical properties at low drying temperatures. Regarding the film properties, some shortcomings have to be considered. The biggest drawback is the dependency on the drying conditions, as these still have a great influence on the final latex film properties. However, with the right functional compound and the right drying conditions, excellent films could be made without the use of any VOC.

The compound responsible for the plasticization effect is water. The used functional compound, the THP, acts as a door or storage to hold the right amount of water and distribute it in the polymer matrix to promote the film formation. For this, it is crucial to understand the water distribution and movement during the film formation. To enable this, during this project a method was developed to monitor the water redistribution during film formation by DSC. The monitoring of the crystallization of bound and unbound water during the latex drying process by DSC helped to understand the redistribution of water domains during the drying process. Two basic mechanisms were recognized. The first one is a steady movement of the water along the polymer matrix and water domain shrinking during the drying. This was the case for the PEGMA and REF latexes. The second occurred for the ITA latex. Here the water domains were set by the ITA, strongly limiting the movement and leading to a splitting of the fixed water domains during the drying process. This second mechanism shows a better water domain distribution, resulting in a stronger MFFT decrease.

The THP concept constitutes a very promising approach, for eliminating VOC from latex paints. However, this concept still needs extended investigations. Important in doing so is the focus on the mechanisms involved in the interaction between water and the polymer matrix, aside from the common film property testing. The latex film formation monitoring by DSC (FFM-DSC) is a powerful tool in carrying out investigations in this direction, and it can complement the information given by other techniques already used in the study of film formation such as small-angle neutron scattering [37] or ^1^H NMR relaxometry [38].

## Figures and Tables

**Table 1:** Formulation of the different latexes.

	REF Latex	MAA Latex	ITA Latex	PEGMA Latex
Compound	wt %	wt %	wt %	wt %
Water	47.75	48.75	48.54	48.75
BA/MMA (40/60 ratio)	50.00	49.5	49.5	49
MAA	-	0.5	-	
ITA	-	-	0.5	
PEGMA	-	-	-	1
SDS	2.00	1.00	1.00	1.00
APS	0.25	0.25	0.46	0.25

**Table 2:** Minimum film-formation temperature (MFFT) and *T*_G_ for temporary hydroplasticizer (THP) latexes, THP amount in the latex in wt % based on the solids content, theoretical water weight % of water saturated polymer calculated with Barrie’s method and theoretical wet *T*_G_ calculated based on [15].

Plasticizer	wt %	*T*_G_ [°C]	MFFT [°C]	Calculated Water % in Water Swollen Polymer	Calculated *T*_G wet_ [°C]
BA/MMA 40/60 Ref.	-	30.3	25 ± 1	3.18	19.0
Methacrylic acid	1.0	30.4	25 ± 3	3.40	18.4
Itaconic acid	1.0	30.6	14 ± 2	3.48	18.3
PEGMA 5005	2.0	28.4	16 ± 1	3.19	17.3

**Table 3:** MFFT, glass transition temperature (*T*_G_) and amount of bound water (%) calculated from DSC, by immediate measurement after the mixture between the polymer and the water.

Plasticizer	wt %	*T*_G_ [°C]	MFFT [°C]	Bound Water % in Water Swollen Polymer
BA/MMA 40/60 Ref.	-	30.3	25 ± 1	13.0
Methacrylic acid	1.0	30.4	25 ± 3	13.8
PEGMA	2.0	28.4	16 ± 1	27.5
Itaconic acid	1.0	30.6	14 ± 2	20.6

**Table 4:** Amount of bound water (g/g polymer), separated in freezing and nonfreezing water calculated from DSC, by measurement after the immersion of the polymer in water for one week.

Plasticizer	Bound Water [g]/ g Polymer	Freezing Bound Water (g/g polymer)	Nonfreezing Bound Water (g/g polymer)
BA/MMA 40/60 Ref.	0.29	0.07	0.22
PEGMA	0.45	0.12	0.33
Itaconic acid	0.33	0.09	0.24

**Table 5:** Correlation between water domain size and freezing temperature [15].

Volume	T_Freeze_
1 cm^3^	−14 °C
1 mm^3^	−24 °C
1 µm^3^	−39 °C
